# Habitat‐and‐Site Dependent Variations on Crustaceans Associated With *Ecklonia radiata* Holdfast Along the South African Wild Coast

**DOI:** 10.1002/ece3.73530

**Published:** 2026-04-27

**Authors:** N. Nkohla, T. S. Dlaza

**Affiliations:** ^1^ Biological and Environmental Sciences Department Walter Sisulu University Mthatha South Africa; ^2^ South African Institute for Aquatic Biodiversity Makhanda South Africa

**Keywords:** biodiversity, gullies, kelp, macrofauna, rock pool, species composition

## Abstract

Crustaceans associated with *Ecklonia radiata* holdfast have long been used as the indicators of environmental change and habitat suitability. Despite this important ecological role, no work has been done to document the spatial variation patterns of crustaceans in the Wild Coast of South Africa. This study examined the site and habitat effects on holdfast‐associated crustacean community composition and documented physical factors underlying observed patterns. Eighty‐four kelp holdfasts produced a total of 1379 individuals among 32 distinct species. Significant positive correlations between total abundance and species richness were prominent in rock pools compared to gullies in most sites. There was a significant interaction effect of site and habitat on species richness. Dwesa gullies had a significantly higher number of species compared to their adjacent rock pools and Xhorha gullies. The total abundance and Shannon diversity differed within Dwesa (gullies vs. rock pools). The composition of crustaceans per holdfast differed significantly between habitats and across sites. Seven clusters were identified, each defined by distinct species. Most gullies were primarily inhabited by barnacles *Amphibalanus venustus*, while isopod *Cymodocella pustulata* dominated the rock pools. Rock pool species were mainly associated with high temperatures and enhanced sodium levels, while the gully species were positively associated with microhabitat characteristics and nitrate. Our findings show that Dwesa gullies had pronounced effects on crustacean biodiversity metrics. Crustaceans in the gullies were mainly driven by nitrate, holdfast, and sediment weight, while rock pool dwellers were characterized by enhanced temperature and sodium.

## Graphical abstract

The crustaceans associated with Ecklonia radiata holdfast varied substantially between rock pools and gullies in different sites. More species inhabited the gullies than the rock pools. This pattern was more prominent in the Dwesa site, while positive associations between abundance and richness were more pronounced in the rock pools.

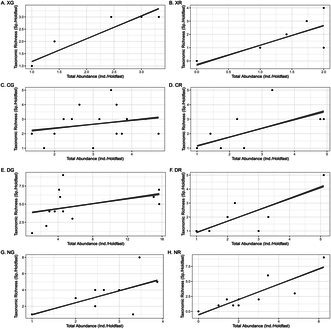

## 1 Introduction

Kelp *Ecklonia radiata* is a macroalga of great ecological significance due to its ability to provide habitat and sustenance for a diverse array of species, ranging from simple microbes to more complex organisms such as mobile fish (Coleman et al. 2011). Commercially valuable animals, such as rock lobsters, octopus, and apex predators like fish and sharks, generally forage for food within the kelp forest (Lankow and Mehta 2023). Invertebrate species form symbiotic relationships with kelp for various purposes such as utilizing their habitat for shelter, depositing their offspring/eggs, benefiting from dispersal through floating, and obtaining nourishment from the macroalgae or the detritus that accumulates in the kelp holdfast (Bolton et al. 2012; Mmonwa 2013; Peterson and Fry 1987).

The faunal communities found in the kelp holdfast have been recognized as potential indicators of human‐induced environmental change (Jones 1973; Moore 1973; Smith 2000). This is attributable to the fact that taxa that live on kelp holdfasts exhibit a predictable pattern of multivariate variation at different scales of an environmental stress gradient (Anderson, Diebel, et al. 2005). In order to assess the impacts of human activities, it is essential to gather baseline information on the response of kelp holdfast‐associated invertebrate populations to natural fluctuations in environmental conditions (Smith 2000). Scientific literature provides evidence that many factors, including temperature, salinity, wave action and exposure, turbidity, depth, and holdfast size, have a moderate influence on the structure of the macroinvertebrates that reside in kelp holdfasts (Jones 1973; Moore 1973; Smith et al. 1996).

Crustaceans are among the dominant macrofaunal groups within kelp holdfast that have been used as the indicators of ecological and environmental change. Crustaceans are suited for this purpose due to their diversity and widespread distribution, as well as their ability to thrive in various environments (Hardege and Fletcher 2022; Navarro‐Barranco et al. 2020).

The distribution of kelp‐associated crustaceans in South Africa is regulated by several factors, including the attributes of kelp holdfasts and the habitat and/or site where they are collected (Katharoyan et al. 2024).

A baseline work on polychaetes associated with *Ecklonia radiata* holdfast between a gully and a rockpool habitat types has shown that the latter harbors a preponderance of species of polychaetes than the former. These findings were linked to calm conditions (Nkohla and Dlaza 2024) and high nutrient deposits in the rockpools compared to the gullies (Nkohla and Dlaza 2025). Although currently, the focus is on crustaceans, it is expected that they would exhibit a similar pattern. The following hypotheses were formulated to investigate the variations in crustacean composition linked to *Ecklonia radiata* holdfasts between rock pools and gullies, as well as across various locations:

*Rock pool habitats exhibit stronger positive correlations between species richness and total abundance than gully habitats, since wave intensity is moderated higher on the shore, where rock pools are mostly found*.

*Local habitat characteristics are more influential than geographical site location in shaping crustacean diversity and composition*.

## 2 Materials and Methods

### 2.1 Study Sites

Dwesa‐Cwebe MPA is co‐managed by the communal property association (CPA) and the Eastern Cape Parks and Tourism Agency (ECPTA), an appointed custodian of marine protected areas in the Eastern Cape. Geographically, this MPA is situated within the Mbashe Local Municipality, across two rural towns, Willowvale and Elliotdale. Historically, before the introduction of marine resources legislation, shellfish harvesting was regulated by the terrestrial act which governed Dwesa Nature Reserve in 1975. In 1991, shellfish exploitation was unlawfully ceased under the Protected Areas Act which targeted the terrestrial reserve (Lasiak 2006). The socio‐political pressure piled by local fishers resulted in reopening Dwesa for multiple uses/controlled harvesting in December 2015 under Section 22A of the National Environmental Management.

Protected Areas Act of 2003 (Act No. 57 of 2003). Before this date, it was a strictly no‐take MPA proclaimed under the Marine Living Resource Act of 1998. Currently, Dwesa‐Cwebe MPA comprises contrasting protection levels known as zones.

### 2.2 Experimental Design

**FIGURE 1:**
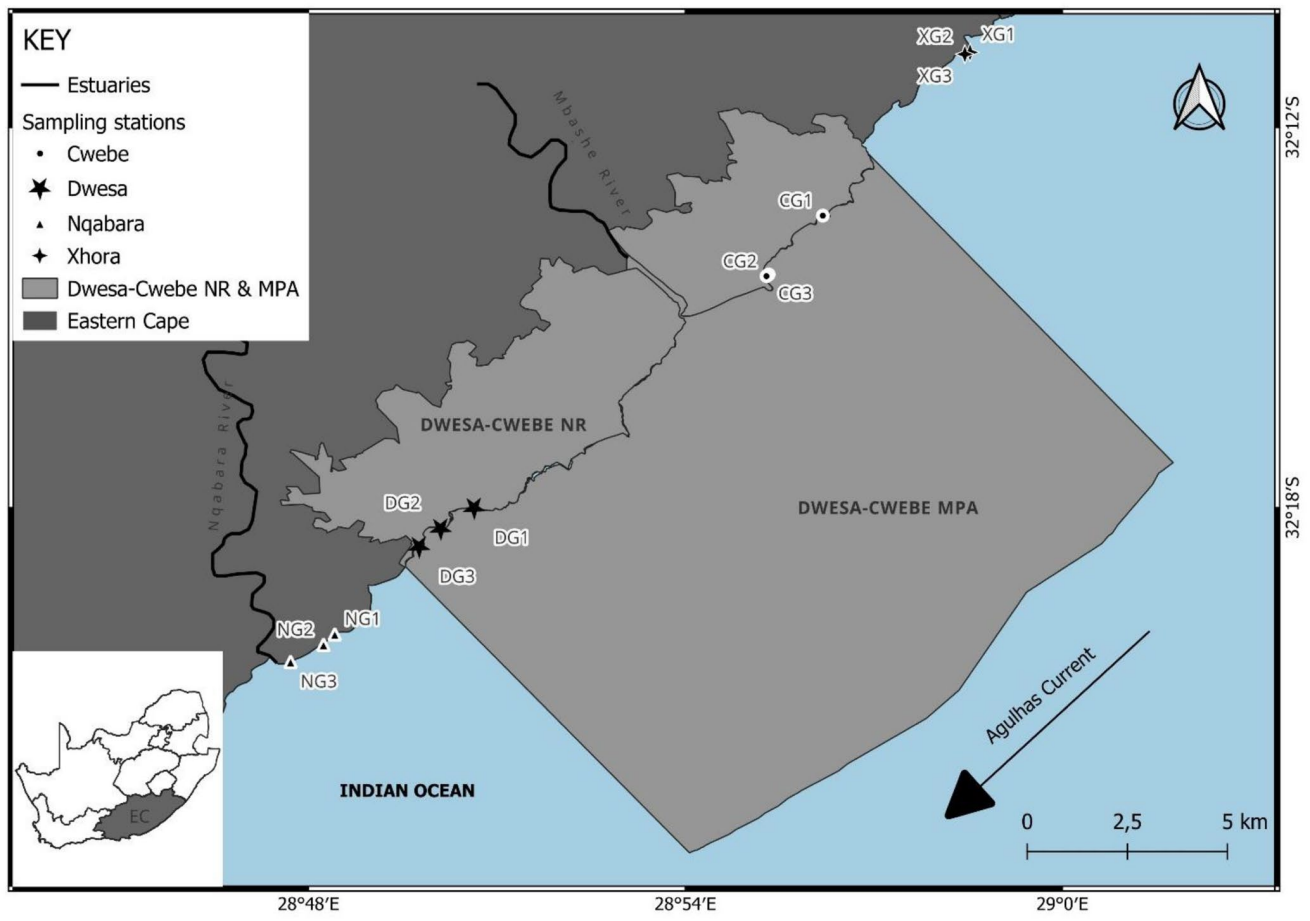
The map of South Africa showing the area of study sites.

The sampling for this study was conducted in four sites: Dwesa and Cwebe MPAs, which are jointly managed by the Eastern Cape Parks and Tourism Agency Parks (ECPTA) as a single unit (Figure 1). Bordering the two MPAs is Nqabarha next to Dwesa MPA and Xhorha close to Cwebe MPA. Dwesa and Cwebe are bisected by a substantial estuarine system called Mbashe. The Mbashe system may act as a geographical barrier for certain kelp‐associated organisms between Nqabarha and Dwesa in the south versus Cwebe and Xhorha in the north. The ecological surveys took place in September–October 2022 on the dates that fell within the low spring tide.

Each of the four sites had three sampling stations, spaced roughly 100 m apart. The treatment consisted of a period of protection lasting more than five years, provided by Dwesa and Cwebe MPA. The control sites were in areas adjacent to the boundaries of the MPAs. The spatial separation among sites ranged from 5 to 20 km (Anderson, Connell, et al. 2005; Anderson, Diebel, et al. 2005). At each of the three sampling stations per site, five replicate holdfasts were sampled randomly, by carefully removing them from the substrate using sharp knives. The stipe was promptly separated from the holdfast to prevent any confusion between the fauna collected from different parts of the sporophyte (Anderson, Connell, et al. 2005, Anderson, Diebel, et al. 2005). The holdfast was then placed in a zip‐lock bag and fixed in 70% Ethanol while transferred to the DSI/NRF‐WSU/SAIAB Joint Marine Biology Laboratory on Coastal Rural Sustainability at Walter Sisulu University.

### 2.3 Laboratory Processing

The holdfast volume in the laboratory was determined by the water displacement method, following the removal of all observable organisms (Blight and Thompson 2008). The holdfast was then split into minute pieces of haptera and rinsed over a 0.5 mm screen (Winkler et al. 2017). The remaining crustacean material on the sieve was meticulously sorted and identified under the dissecting microscope, employing the standard taxonomic keys (Branch 2018; Day 1969; Griffiths 1976; Kensley 1978), recent publications that provide detailed descriptions of the species (Milne and Griffiths 2013), and the expert opinions of Charles Griffiths from the University of Cape Town and Olwethu Duna formally with Anchor Environmental Consultant. The study evaluated both alpha diversity (species richness) and beta diversity (composition), as well as the relative abundance within and between sites. This was done by creating a matrix that enumerated the species present and recorded zero for species that were missing in each holdfast. The wet weight of the sediment per holdfast was measured using an electronic weighing balance (Teagle and Smale 2018).

### 2.4 Data Analyses

All the statistical analyses and data visualization plots were generated from Rstudio (Team 2021). Data were explored for homogeneity and normality using the fligner.test() and shapiro.test() functions in R, respectively (Bates et al. 2007). After deviation from the normality and homogeneity, non‐parametric tests were conducted to analyze our datasets. To test our first hypothesis, we ran a spearman rank correlation coefficient to explore the relationship between the total abundance and species richness between rock pools and gullies across different sites. For the second hypothesis, a two‐way factor analysis of variance (ANOVA) based on generalized least squares was fitted to analyze the site and habitat interaction effect on the response variables (species richness, total abundance, Shannon diversity, and J'Pielou evenness). A Holm‐adjusted pairwise comparisons of estimated marginal means (Satterthwaite df) was conducted to ascertain the difference among the sites and habitats. Permutational Multivariate Analysis of Variance (PERMANOVA) was run to discriminate the composition of taxa between and within habitats and sites. To validate the model results, a variance among groups was tested with the betadisper function. A significant dispersion was observed between habitats, and a modified PERMANOVA model (betadisper = TRUE) that considers the variance among groups was fitted (Oksanen 2017). Where a significant difference in species composition was detected between groups, a PERMANOVA pairwise test was conducted to determine sites that were significantly different from each other (Martinez Arbizu 2020).

The principal coordinate analysis (PCoA) technique was employed to visually represent the spatial structure of crustacean communities and analyze the patterns of variation between rock pools and gullies across sites. Additionally, it identified the indicator species of each cluster using the Indicspecies package in R (De Cáceres et al. 2010). Finally, a redundancy analysis (RDA) model was fitted to identify the important environmental drivers for the observed patterns of variation in crustacean species composition through a vegan package in R. The biological data was Hellinger transformed. The environmental data included categorical (site and habitat) and numerical variables (Table 1). The envfit() function was fitted to select the main numerical variables responsible for the observed variation patterns in crustacean species composition in ordination space.

**TABLE 1:** Environmental parameters linked to the sampling locations of *Ecklonia radiata* holdfasts.

Environmental parameters	XG	XR	CG	CR	DG	DR	NG	NR
Sediment weight (g)	9.42 (5.60)	6.38 (5.58)	18.89 (16.07)	12.51 (6.91)	31.68 (21.91)	7.41 (8.77)	20.51 (11.85)	21.59 (11.85)
pH	7.56 (0.13)	7.57 (0.06)	7.50 (0.04)	7.63 (0.06)	7.23 (0.55)	7.64 (0.21)	7.44 (0.16)	7.76 (0.09)
Temperature (°C)	20.37 (0.63)	20.65 (1.0)	19.57 (0.69)	20.05 (1.71)	18.89 (0.25)	18.56 (0.44)	19.53 (0.30)	20.91 (0.34)
Sodium (μg L^−1^)	113.81 (6.51)	112.5 (2.64)	98.67 (1.95)	101 (7.43)	96.08 (3.15)	105.66 (5.27)	110.62 (27.49)	96.6 (0.97)
Nitrate (μg L^−1^)	158.10 (3.25)	163.33 (3.51)	166.67 (4.88)	168.18 (12.50)	155 (13.82)	122.89 (27.68)	143.08 (39.02)	91.70 (0.48)
Depth (cm)	56.43 (5.86)	38 (8.43)	49.63 (19.45)	62.95 (2.29)	64.67 (33.86)	68.27 (18.89)	103.31 (75.56)	42.90 (6.28)
Salinity	17.74 (0.10)	17.75 (0.05)	17.86 (0.13)	17.78 (0.14)	17.43 (0.99)	18.36 (0.42)	17.78 (0.31)	18.0 (0)
Holdfast volume (mL)	54.71 (25.86)	51.30 (24.25)	81 (57.14)	52.27 (30.77)	108.75 (67.76)	40.56 (43.04)	96 (39.94)	80 (46.67)
Haptera wet weight (g)	33.86 (20.46)	22.50 (14.78)	45.28 (37.12)	31.80 (15.43)	59.67 (42.09)	16.94 (16.17)	35.96 (20.08)	36.60 (20.83)

*Note:* The numbers outside the parentheses are the means, and those inside are standard deviations.

Abbreviations: CG, Cwebe gullies; CR, Cwebe rock pools; DG, Dwesa gullies; DR, Dwesa rock pools; NG, Nqabarha gullies; NR, Nqabarha rock pools; XG, Xhorha gullies; XR, Xhorha rock pools.

## 3 Results

### 3.1 Species Richness Versus Total Abundance in Different Locations

**FIGURE 2:**
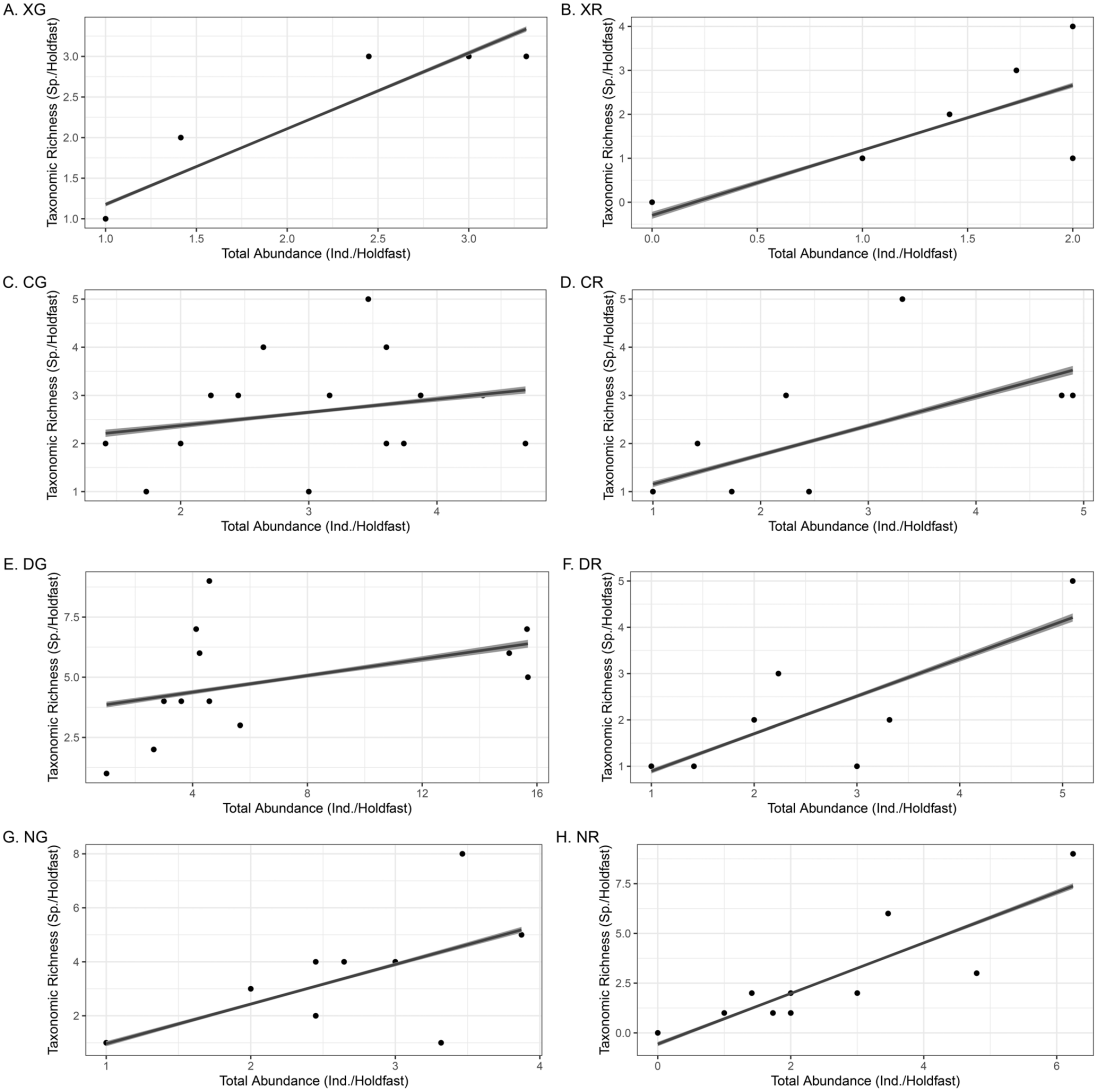
Scatterplot showing the relationship between species richness and total abundance in different sites and habitats. Species richness versus total abundance in different locations (labeled A–H). The locations were abbreviated as follows. Site: X, Xhorha; C, Cwebe; D, Dwesa; N, Nqabarha. Habitat type: G, Gully; R, Rock pool.

Eighty‐four kelp holdfasts yielded a total of 1379 individuals belonging to 32 different species. In Xhorha gullies, there were about 31 individuals distributed across 10 species, 10 genera, 8 families, 3 orders, and 2 classes. A spearman rank correlation coefficient showed a strong positive correlation between the number of species and total abundance in this habitat (Figure 2A; *r* = 0.9; *p* < 0.001). On the other hand, Xhorha rock pools had a total of 18 individuals distributed across 11 species, 11 genera, 11 families, 4 orders, and 2 classes. There was also a strong positive relationship between the number of species and the number of individuals (Figure 2B; *r* = 0.8; *p* < 0.001) this habitat. A total of 154 individual distributed across 13 species, 11 genera, 11 families, 6 orders, and 4 classes were recorded in Cwebe gullies. Conversely, 83 individuals, 14 species, 14 genera, 11 families, 6 orders, and 3 classes were recorded in Cwebe rock pools. Interestingly, in this habitat type, there was a positive relationship between the species richness and total abundance (Figure 2D; *r* = 0.7; *p* = 0.03). Dwesa gullies included a total of 856 individuals, distributed among 14 species, 14 genera, 12 families, 6 orders, and 3 classes. On the other hand, Dwesa rock pools had a total of 60 individuals, distributed across 17 species, 14 genera, 13 families, 6 orders, and 3 classes. Within Dwesa rock pools, the total abundance was positively correlated with species richness (Figure 2F; *r* = 0.7; *p* = 0.02). Nqabarha gullies had a total of 80 individuals, belonging to 17 species, 15 genera, 13 families, 4 orders, and 2 classes. A positive association between total abundance and species richness was detected in this habitat type (Figure 2G; *r* = 0.7; *p* = 0.003). On the other hand, in Nqabarha rock pools, there were 97 individuals distributed across 19 species, 19 genera, 19 families, 7 orders, 4 classes. There was a strong positive correlation between the number of individuals and species richness (Figure 2H; *r* = 0.9; *p* < 0.001).

### 3.2 Biodiversity Patterns of Kelp Holdfast‐Associated Crustaceans in Different Locations

**FIGURE 3:**
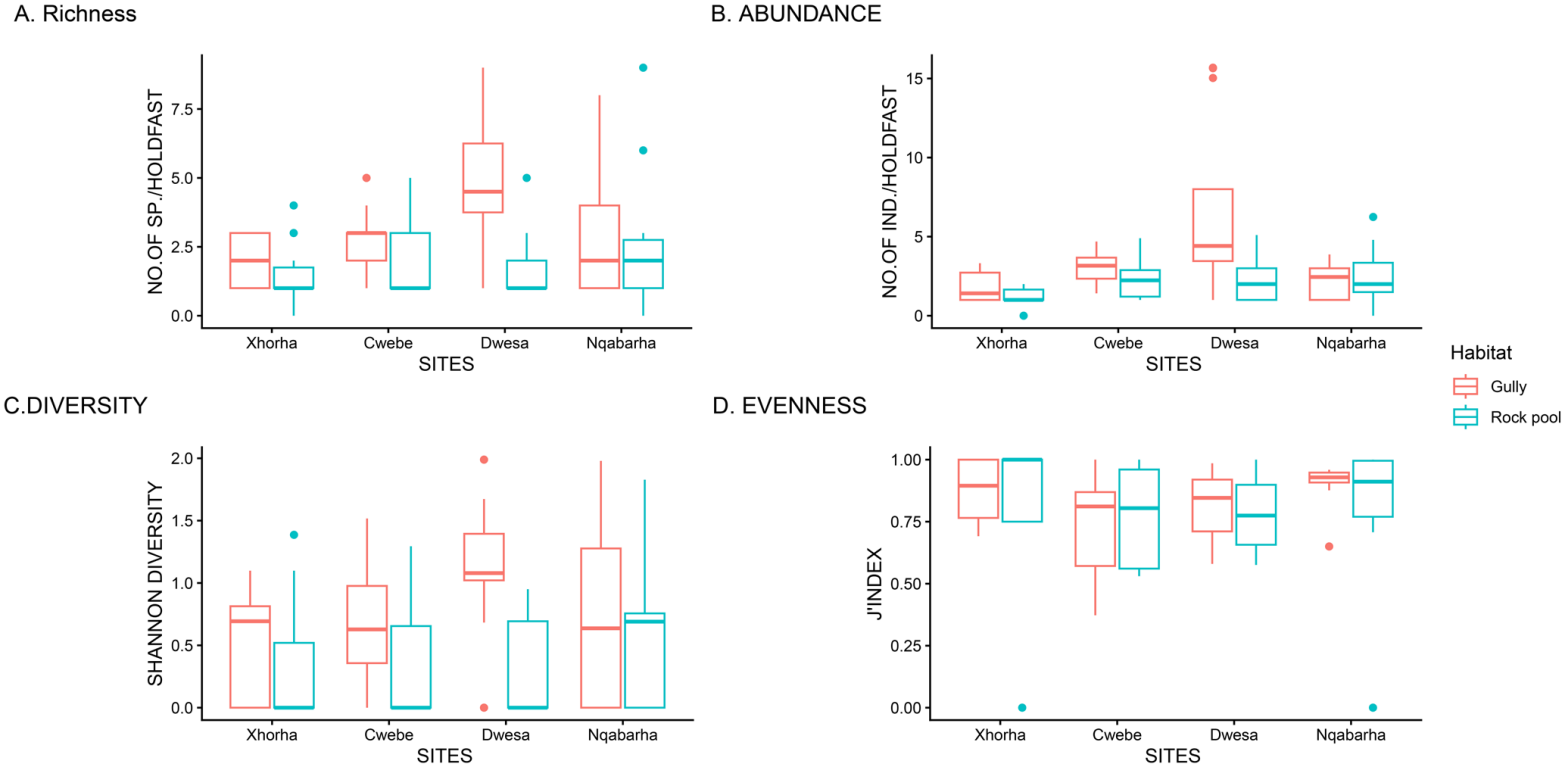
Boxplots showing the differences in biodiversity metrics between sites and habitats. The y‐axis is the response metric, *x*‐axis is the site, and the fill/key is the habitat type. A, Species richness; B, Total abundance; C, Shannon diversity; D, J'Pielou evenness index.

The number of species increased varied between sites and habitat types (Figure 3). Dwesa site (04 ± 2.4) had on average the highest number of species, while Xhorha (02 ± 1.0) had the lowest number of species. Pertaining to the habitat types the species richness was generally low in the rock pools, compared to the gullies. Xhorha rock pools (1.7 ± 1.1) had lowest mean species richness compared relative to adjacent gullies (02 ± 1.0). Dwesa gullies (05 ± 2.3) had the highest number of species compared to adjacent gullies. A two‐way factorial ANOVA via generalized least squares (GLS) revealed a statistically significant interaction effect of site and habitat for species richness (GLS two‐way ANOVA: *F*‐value = 241.11; df = 3; *p* = 0.04). Furthermore, of the main effects, habitat was the only factor to exhibit a significant effect (*F* = 6.94; df = 1; *p* = 0.01). A post hoc test revealed that a notable difference was only detected between a rock pool and gully (*p* < 0.001) in Dwesa, and among Xhorha and Dwesa gullies (*p* = 0.01).

Dwesa (4.8 ± 4.7) had the highest mean number of individuals, while Xhorha (2.4 ± 1.5) had the least (Figure 3B). Pertaining to the habitats, Xhorha gullies (1.9 ± 1.0) had slightly more individuals on average compared to adjacent rock pools (1.2 ± 0.6). On the other hand, Dwesa gullies (6.7 ± 5.4) had the highest number of individuals on average relative to adjacent rock pools (2.2 ± 1.4). There was a statistically significant site × habitat interaction for crustacean abundance (GLS two‐way ANOVA: *F* = 2.89; df = 3; *p* < 0.04). Site was the only factor to exhibit the significant effect (*p* < 0.001). In particular, the total abundance of crustaceans was significantly higher in Dwesa gullies compared to the Dwesa rock pools (*p* = 0.02).

The Shannon diversity varied within sites, and among habitat types (Figure 3D). The highest mean Shannon diversity was shared by both Dwesa and Cwebe (0.7 ± 0.6), whereas the lowest mean diversity was recorded in Xhorha (0.4 ± 0.5). In relation to habitats Xhorha rock pools (0.4 ± 0.6) were slightly lower on mean Shannon diversity compared to adjacent gullies (0.5 ± 0.5). On the other hand, Dwesa gullies (1.1 ± 0.5) were had the higher Shannon diversity than adjacent rock pools (0.3 ± 0.4). Hence, a statistically significant habitat effect (GLS two‐way ANOVA: *F* = 11.06 df = 1; *p* < 0.001) was detected. The substantial difference was only detected between Dwesa gullies and Dwesa rock pools (Figure 3C; *p* < 0.001).

No statistically significant difference was detected on the species evenness.

### 3.3 Crustacean Community Structure Associated With Kelp Holdfasts Across Sites and Habitats

**FIGURE 4:**
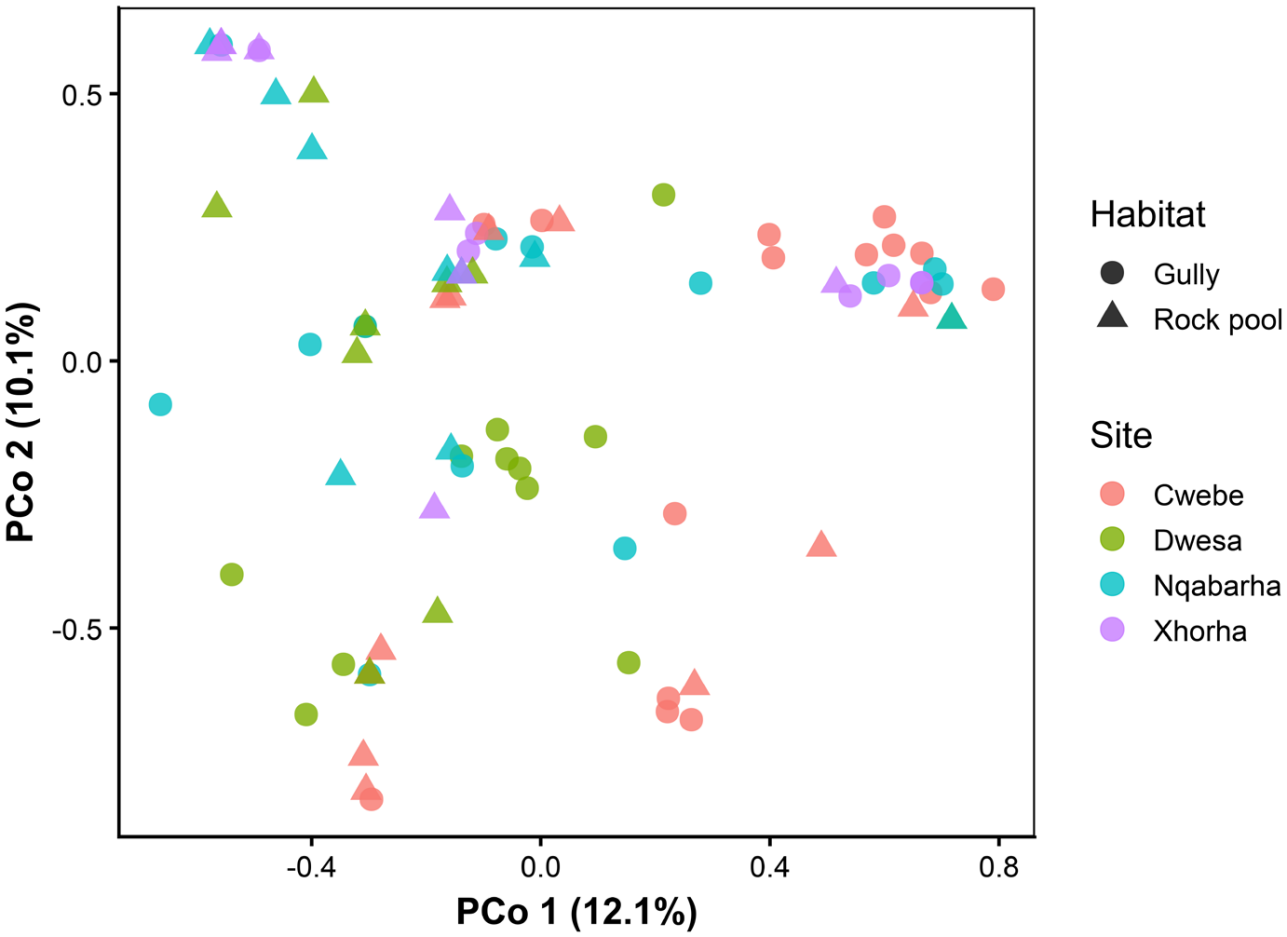
Principal coordinate analysis (PCoA) showing the clustering of the crustaceans associated with kelp holdfast based on Bray‐Curtis dissimilarities. Points represent samples, colored by sites, and shaped by habitats.

The crustacean composition differed significantly between sites. The habitat influence on crustacean composition was consistent across sites (Figure 4). The unique combination of clusters of species between habitats and sites was statistically significant (Table 2). Although both site and habitat were significant drivers of crustacean composition, no evidence of interaction was detected.

**TABLE 2:** Permutational multivariate analysis of variance (PERMANOVA) showing the variance in crustacean community composition across sites and habitat types.

Predictors	Degrees of freedom	Sum of squares	Pseudo‐*F*	*p*
*Permanova*				
Site	3	35,064	3.41	0.001
Habitat	1	12,570	3.66	0.001
Site × Habitat	3	4424.7	1.29	0.148
Residual	76	260.82		
Total	83	323.67		

Pairings	*t*‐value	Unique permutations	*p*
*Permanova pairwise test*			
Cwebe versus Dwesa	2.21	998	0.001
Cwebe versus Nqabarha	1.99	999	0.001
Cwebe versus Xhorha	2.00	999	0.003
Dwesa versus Nqabarha	1.37	998	0.060
Dwesa versus Xhorha	2.06	999	0.001

The principal coordinate analysis (PCoA) was able to explain at least 20% of the total variation, with PCO 1 explaining 12.1%, while PCO 2 explained 10.1%. There were clear separations between all the sites, except Dwesa and Nqabarha. Cwebe formed a distinct cluster on the right side of the plot, while Dwesa clustered more centrally. Xhorha and Nqabarha exhibit no clear ecological structuring, either than overlapping with other clusters. The bottom of the plot was mainly occupied by Dwesa and Cwebe (Figure 4). The habitat types exhibited distinct ecological structuring in ordination space. The rock pools clustered higher on the PCoA 2 axis, whilst their gully counterparts occupied the PCoA 1 axis, although they formed a substantial cluster at the centre of the ordination space. Generally, there was an overlap in the crustacean composition between habitat types from the same site. Contrarily to the general pattern, crustacean assemblages from the gullies clustered separately from their adjacent rock pool counterparts in the Cwebe site (Figure 4).

### 3.4 Indicator Species for Different Locations

The species that may have influenced the distinction among the clusters are presented below (Table 3). An indicator species for CG cluster was *Parisocladus perforatus* and perhaps the one that has separated this cluster from most clusters. In cluster DG, a grazing tanaid *Zeuxoides helleri* and wood‐eating *Limnoria quadripunctata* was also highly influential in this cluster in terms of frequent occurrences. The clusters DG and NR were characterized by predatory isopods *Paranthura punctata* and *Cirolana venusticauda* . On the other hand, cluster CG + DG + NG + XG, which comprises mainly all the gullies, was dominated by the filter‐feeding barnacle *Amphibalanus venustus*. Interestingly, cluster DG + DR + NG + NR + XR, which mainly consisted of rock pools, with DG and NG being the only exceptions, was characterized by the herbivorous isopod *Cymodocella pustulata* .

**TABLE 3:** Indicator Value Analysis (IndVal) of holdfast‐associated crustaceans across locations.

Location	Species	Ecological role	IndVal	*p*
CG	*Parisocladus perforatus*	Grazer	0.71	0.01
DG	*Limnoria quadripunctata*	Grazer	0.87	0.01
DG	*Zeuxoides helleri*	Grazer	0.77	0.01
DG + NR	*Paranthura punctata*	Predator	0.60	0.01
DG + NR	*Cirolana venusticauda*	Predator	0.58	0.05
CG + DG + NG + XG	*Amphibalanus venustus*	Filter feeder	0.77	0.01
DG + DR + NG + NR + XR	*Cymodocella pustulata*	Grazer	0.63	0.02

*Note:* Abbreviated locations denote sampling sites and a habitat type. Site: X, Xhorha; C, Cwebe; D, Dwesa; N, Nqabarha. Habitat type: G, Gully; R, Rock pool.

**FIGURE 5:**
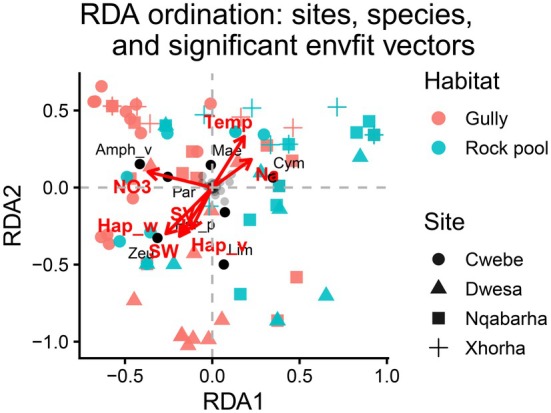
A redundancy analysis (RDA) plot showing the important environmental variables that shaped that community composition of holdfast‐associated crustaceans between sites and habitats. Habitat types were differentiated by colors. The pink circles represent gullies, while blue circles represent rock pools. Site were distinguished by different shapes. Circle represented Cwebe, while a triangle represented Dwesa, square represented Nqabarha, and a cross represented Xhorha. Inside the ordination space, there are acronyms of crustaceans and the environmental variables (see Table S1 for full names). The arrows denoted the direction and strength of the association between biological and environmental data.

### 3.5 Environmental Drivers of Crustacean Species Composition

The redundancy analysis (RDA) model was able to explain 14.93% of the total variance. The first two constrained axes, RDA 1 and RDA 2 accounted for 6.21% and 5.71% respectively: explaining about 11.92% of the total variance (Figure 5). The positive side of RDA 1 was associated with high temperatures and sodium levels, suggesting drier conditions. As such, this axis was characterized by the rock pool habitats (right side). Crustacean species that positively correlated with these conditions were *Maera sp*, *Cymodocella pustulata* , *Parisocladus perforatus* , and *Limnoria quadripunctata* (see Table S1 for full names and corresponding codes). The left (negative) side of the RDA 1 axis was characterized by the gully habitat. This clustering was driven by high levels of nitrate. The crustacean species that thrived well in these conditions included *Amphibalanus venustus* and *Paranthura punctata* . On the other hand, the negative side of RDA 2 was mainly driven by haptera and sediment weight and volume. The tanaid *Zeuxoides helleri* exhibited a strong association with haptera weight and sediment volume. As far as the sites are concerned, there were numerous overlaps, particularly across Cwebe, Dwesa, and Nqabarha. The only site to display a clear trend was Xhorha, that was characterized by the higher levels of temperature and sodium concentration. Both site (RDA: df = 1; *F*‐value = 3.48; *p* = 0.001) and habitat (RDA: df = 3; *F*‐value = 3.58; *p* = 0.001) were the significant drivers of the variation pattern in crustacean species composition. Furthermore, the significant environmental drivers were haptera wet weight (*r* ^2^ = 0.17; *p* = 0.001), nitrate (*r* ^2^ = 0.14; *p* = 0.005), sediment wet weight (*r* ^2^ = 0.12; *p* = 0.007), sediment volume (*r* ^2^ = 0.11; *p* = 0.012), sodium (*r* ^2^ = 0.09; *p* = 0.014), and haptera volume (*r* ^2^ = 0.08; *p* = 0.03).

## 4 Discussion

The total abundance and species richness in our results were 16.22/holdfast and 0.32/holdfast, respectively. This ratio indicates that for every 10 holdfasts, there will be a minimum of 162 individuals and 32 species of crustaceans present in our study sites. The yield indicates a significant abundance, demonstrating the importance of holdfasts as microhabitats (Smith et al. 1996). A greater density of individuals within a given area enhances the probability of species interactions, including competition, predation, and symbiosis, thereby affecting community dynamics and augmenting ecosystem complexity (Vanni et al. 2009). The low ratio of species richness per holdfast indicates that not all holdfasts harbor unique species. A low average of species per holdfast indicates high beta diversity, driven by a significant species turnover rate among holdfasts. This indicates that each holdfast uniquely contributes to the overall diversity. Factors contributing to microhabitat variability include individual holdfast complexity, characterized by the arrangement of haptera, presence of crevices, sediment accumulation, holdfast shape, habitat exposure to wave action, patch sizes, density of the kelp forest, and surrounding ecosystems (Velasco‐Charpentier et al. 2021). The relationship between total abundance and species richness varied within and between locations. However, strong positive associations were generally frequent in rock pools across all sites. These results suggest that in terms of habitat suitability, rock pools offer comfortable living conditions, mainly due to moderated wave intensity capturing suspended particles compared to the gullies (Gonçalves et al. 2023). Another plausible explanation for this would be the reduced predation effect in rock pools since these habitats are only periodically covered by the sea water (Chang and Todd 2023). On the other hand, the gullies are directly connected to the water column, since these structures are only a furrow that extends to the rock shores but are often submerged with water. The water can only recede during the spring low tide, but not all of it. This implies that gullies are more accessible to predators than rock pools (Thompson et al. 2010).

### 4.1 Habitat Type Exerts Greater Influence Than Geographic Location on Crustacean Biodiversity Associated With Kelp Holdfasts

Our univariate statistical results showed that the Dwesa site had significantly higher species richness compared to Xhorha. The differences between these sites could be attributed to holdfast characteristics. Holdfasts collected in Dwesa were generally larger and contained a high accumulation of sediment compared to those sampled in Xhorha. The effect of holdfast and sediment on biodiversity patterns is well supported by literature (Anderson, Diebel, et al. 2005; Baldrich et al. 2024; Nkohla and Dlaza 2024, 2025; Ronowicz et al. 2018; Smith et al. 1996). Dwesa gullies were generally associated with higher ecological indices relative to adjacent rock pools. This pattern could be attributed to enhanced nutrients and stable environmental conditions in the gullies compared to longer periods of exposure to sunlight in the rock pools. As portrayed by the RDA, rock pools are linked to drier conditions and elevated salinity levels, indicative of a high evaporation rate (Legrand et al. 2018). The general trend in our results is that the habitat effect is consistent across all the sampled sites. Dwesa gullies are biodiversity hotspots, while Xhorha rock pools are very poor in terms of biodiversity. Be that as it may, these results need to be interpreted with caution due to small sample size.

The composition of crustaceans within *Ecklonia radiata* holdfast was unique for each site. This variation in composition patterns suggests a high turnover rate between sites. Unique clusters between sites could be indicative of site‐specific heterogeneous features, probably driven by local environmental conditions (Nkohla and Dlaza 2025). The habitat effect was prominent in species composition and explained most of the observed variation patterns. Most rock pools tended to form aggregations (right and centre of PCoA ordination space), regardless of the site. Similarly, the gullies were distributed on the left side of the ordination space. This pattern was explicitly shown in the RDA space, where the right side was mainly dominated by the rock pools and was positively associated with increased temperatures and sodium. These conditions are common in intertidal rock pools (Legrand et al. 2018). Thus, the crustaceans occupying the rock pools will have adaptations to cope with dynamic conditions.

The principal coordinate analyses which have captured more than 20% of the variation in crustacean composition within kelp holdfasts in different sites and across different habitat types augmented the PERMANOVA output by showing the clustering driven by both the site and a habitat type. The clustering on the NMDS demonstrates a dominant habitat influence on the crustacean composition within kelp holdfasts (Nkohla and Dlaza 2024; Walls et al. 2016).

The Dwesa and Cwebe gullies were dominated by the isopods *Limnoria quadripunctata* and *Parisocladus perforatus* , respectively. Isopods *Limnoria quadripunctata* and *Parisocladus perforatus* are moisture‐dependent, thus require a habitat that possesses a greater water retention. Gullies provide optimum conditions for organisms preferring to be immersed in water for longer periods, hence the dominance of *Limnoria quadripunctata* and *Parisocladus perforatus* on kelp growing in the gullies, relative to kelp growing in the rock pools (Kensley 1978). On the other hand, the dominance of tanaid *Zeuxoides helleri* in the Dwesa gullies could be attributed to their low dispersal ability (Esquete et al. 2012). Tanaids, like most small‐bodied animals, tend to have limited movement; therefore, since it is highly possible that the parent population of *Ecklonia radiata* in Eastern Cape is in the gullies, with the rock pools only receiving sporophyte seeds dispersed from the former. This implies that the tanaids are most likely to almost every time settle in the gullies where the natal population of tanaids is based. The occurrence of predatory isopods *Paranthura punctata* and *Cirolana venusticauda* in Dwesa gullies and Nqabarha rock pools indicates a substantial availability of their prey, presumably small benthic invertebrates. This pattern may signify increased local productivity in these ecosystems relative to other locations, maybe influenced by advantageous environmental circumstances that sustain a diverse food web (Cowles et al. 2009). The barnacle *Amphibalanus venustus* dominated the holdfast space in the gullies, reinforcing the environmental filtering caused by harsh wave action, as well as the availability of suspended organic matter and plankton coming offshore for the filter‐feeders in the holdfast (Reustle et al. 2023). The conditions in the gullies force the organisms to attach firmly to the substrate and be able to feed through capturing suspended particles in the water column, rather than grazing and risking being dislodged (Hargrave et al. 2004). Furthermore, despite living in the habitat frequently visited by predators, barnacles are rarely eaten and are well protected by their hard calcareous shells (Liang et al. 2019). Furthermore, gullies present favorable environmental conditions for high survival of barnacles relative to rock pools, which are characterized by elevated temperatures and salinity levels, linked to a high mortality rate of *Amphibalanus* species (Buasakaew et al. 2021). The grazing isopod *Cymodocella pustulata* was more prominent in the rock pools than in the gullies. The only time they dominated the gullies was in Dwesa and Nqabarha sites. These findings imply that rock pools, with laminar water flow, are suitable for grazing feeding mode, hence the *C. pustulata* dominance. The *C. pustulata* do not directly feed on kelp but graze on epiphytes associated with the kelp holdfast (Nkohla and Dlaza, unpublished). Their dominance in Dwesa and Nqabarha gullies could be attributed to the topography, which is similar in these sites (Nkohla and Dlaza, unpublished).

Hypothesis 1 was corroborated by our data, which demonstrated a positive correlation between total abundance and species richness in the rock pools. This trend was anticipated due to the tranquil conditions prevalent in rock pools, thereby fostering a more stable ecosystem compared to gullies. Similarly, Hypothesis 2 was also supported by our data. Habitat type emerged as the significant factor, with gullies generally exhibiting higher biodiversity, and the species in this habitat were positively associated with holdfast characteristics and nutrients. These findings also contrast those reported for the polychaetes at the same sites, where rock pools exhibited more species richness than the gullies (Nkohla and Dlaza 2024). Most species, including *Parisocladus perforatus, Limnoria quadripunctata, Paranthura punctata, Cirolana venusticauda*, and *Amphibalanus venustus*, displayed strong affinities for gullies, whereas only *Cymodocella pustulata* was associated with rock pools.

The main limitation to this work is the low sampling size; thus, a precaution needs to be applied when interpreting our findings.

## Author Contributions

**N. Nkohla:** conceptualization (equal), data curation (equal), formal analysis (equal), investigation (equal), methodology (equal), project administration (equal), software (equal), visualization (equal), writing – original draft (equal), writing – review and editing (equal). **T. S. Dlaza:** conceptualization (equal), data curation (equal), formal analysis (equal), funding acquisition (equal), investigation (equal), project administration (equal), resources (equal), supervision (equal), writing – review and editing (equal).

## Funding

I would like to express gratitude to the National Research Foundation (NRF) for funding my PhD project, which has led to the production of this work. The grant number: PMDS230727138925.

## Conflicts of Interest

The authors declare no conflicts of interest.

## Supporting information


**Table S1:** Species list table showing the full names of species and environmental factors and the associated codes.

## Data Availability

Data for this work is made available on the following database: Nkohla and Dlaza (2026).
