# Supplementary material for: Habitat‐and‐Site Dependent Variations on Crustaceans Associated With Ecklonia radiata Holdfast Along the South African Wild Coast
Source: Ecol Evol. 2026 Apr 27;16(4):e73530. doi: 10.1002/ece3.73530 (PMC13112080; doi:10.1002/ece3.73530)
Supplement: Supplementary file 1 — Table S1: Species list table showing the full names of species and environmental factors and the associated codes. [file ECE3-16-e73530-s001.docx]

**Supplementary material**

**Table S1:** Species list table showing the full names of species and environmental factors and the associated codes.

| **Species Name** | **Code** | **Physical factor** | **Code2** |
| --- | --- | --- | --- |
| *Maera sp* | *Mae* | pH | pH |
| *Parisocladus perforatus* | *Par* | Temperature | Temp |
| *Joeropsis stebbingi* | *Joe* | Na | Na |
| *Amphibalanus venustus* | *Amph_v* | NO3 | NO3 |
| *Leucisca squalina* | *Leuc* | Depth | Dep |
| *Tanystylum brevipes* | *Tan* | Salinity (‰) | Sali |
| *Cirolana venusticauda* | *Cir_v* | Holdfast volume (ml) | HV |
| *Eriphia sebana* | *Eri* | Haptera volume (ml) | Hap_v |
| *Ostracoda* | *Ost* | Haptera weight (g) | Hap_w |
| *Zeuxoides helleri* | *Zeu* | Sediment volume (ml) | SV |
| *Ampelisca cf. spinimana* | *Amp* | Sediment wet weight (g) | SW |
| *Limnoria quadripunctata* | *Lim* |  |  |
| *Paranthura punctata* | *Par_p* |  |  |
| *Amphibalanus amphritite* | *Amph_a* |  |  |
| *Cymodocella pustulata* | *Cym* |  |  |
| *Mesanthura catenula* | *Mes* |  |  |
| *Haliophasma macrurum* | *Hal* |  |  |
| *Melita sp* | *Mel* |  |  |
| *Gnathia spp* | *Gna* |  |  |
| *Lysianassa sp* | *Lys* |  |  |
| *Lysianassa ceratina* | *Lys_c* |  |  |
| *Caprella equilibra* | *Cap* |  |  |
| *Dynamenella dioxus* | *Dyn_d* |  |  |
| *Stenetrium spp* | *Ste* |  |  |
| *Podocerus sp* | *Pod* |  |  |
| *Exosphaeroma varicolor* | *Exo* |  |  |
| *Ianiropsis palpalis* | *Ian* |  |  |
| *Ischryomene huttoni* | *Isch* |  |  |
| *Guinusia chabras* | *Gui* |  |  |
| *Tetraclita serrata* | *Tetr* |  |  |
| *Pilumnus minustus* | *Pil* |  |  |
| *Cymadusa filosa* | *Cym_f* |  |  |
